# Effectiveness of bone substitute materials in opening wedge high tibial osteotomy: a systematic review and meta-analysis

**DOI:** 10.1080/07853890.2022.2036805

**Published:** 2022-02-15

**Authors:** Tao Bei, Liping Yang, Qiulin Huang, Jiaheng Wu, Junting Liu

**Affiliations:** a Department of Orthopaedics Trauma and Hand Surgery, The First Affiliated Hospital of Guangxi Medical University, Nanning, China; b Guangxi Medical University, Nanning, China; c Guangxi Engineering Center in Biomedical Materials for Tissue and Organ Regeneration, Guangxi Collaborative Innovation Center for Biomedicine, Guangxi Key Laboratory of Regenerative Medicine, The First Affiliated Hospital of Guangxi Medical University, Nanning, China; d Department of Acute Care Surgery, The First Affiliated Hospital of Guangxi Medical University, Nanning, China

**Keywords:** Knee osteoarthritis, opening wedge high tibial osteotomy, bone substitute material, graft

## Abstract

**Background:**

A meta-analysis of eligible studies was performed to evaluate the effectiveness of bone substitute materials (BSMs) in opening wedge high tibial osteotomy (OWHTO) for knee osteoarthritis.

**Methods:**

A systematic review and meta-analysis were conducted according to the Preferred Reporting Items for Systematic Reviews and Meta-Analysis (PRISMA). A comprehensive literature search was performed, and studies comparing BSM with bone graft (BG) and without bone graft (WG) were included. The Cochrane risk of bias tool (version 1.0) and Risk of Bias in Non-randomized Studies of Interventions (ROBINS-I) tool were used to assess the risk of bias for randomized controlled trials (RCTs) and non-randomized studies (NRSs), respectively. The outcomes measured were the osteotomy gap size, the occurrence rates of non-union and lateral hinge fractures, knee functional score, infection and the Visual Analogue Scale (VAS). The quality of evidences was evaluated by Grades of Recommendation, Assessment, Development and Evaluation (GRADE) Working Group system.

**Results:**

Five RCTs and eight NRS including 769 participants were included in our meta-analysis. The BSM group had a larger osteotomy gap size than the control group (MD: 0.41 mm, 95% confidence interval (CI): [0.06, 0.76], *p*=.02, *I*^2^=0%), with a significant difference. No significant difference was found between BSM and control group in main analysis in terms of bone non-union, but with a higher non-union rate when BSM combined with long locking plate was used. No significant differences were found in other outcome measures except for VAS from NRS subgroup. The quality of evidence for outcomes was low.

**Conclusions:**

BSM combined with locking plate techniques offers a safe and efficient alternative option in OWHTO for osteotomy gap larger than 10 mm, but be aware of the possibility of bone non-union. Given the inherent heterogeneity and low quality of the included studies, future well-designed RCTs are essential to verify the findings.

**KEY MESSAGE**
- The treatment of the osteotomy gap is still controversial.
- BSM combined with a locking plate offers a safe and efficient alternative option for OWHTO with an over 10 mm of osteotomy gap over 10 mm.
- Due to the inherent heterogeneity and low quality of the included studies, the results should be cautiously interpreted in clinical practice.

## Introduction

Knee osteoarthritis (KOA) is a common joint degenerative disease and a leading cause of pain and disability in older people. Knee malalignment is associated with the risk of KOA progression [1]. Opening wedge high tibial osteotomy (OWHTO) with medial plate fixation is a well-established surgical procedure to treat early-stage KOA, especially for varus knee [2,3]. However, a wedge-shaped defect will be created when the osteotomy space is opened, which could be left to heal or grafted with either a bone or a bone substitute material (BSM) [4]. The most common complications of OWHTO are the risk of delayed bone healing, non-union, lateral hinge fractures (LHFs) and loss of correction.

Traditionally, the osteotomy site is filled within bone graft (BG) including autograft (AU) and allograft (AL). AU is considered the “gold standard” for bone regeneration procedure [5]. However, the BG harvesting procedure is associated with prolonged operative time, donor morbidity, pain, haematoma and infections. Alternatively, AL can eliminate these complications, but it carries a risk of disease transmission [6] and is less effective at stimulating bone healing. BSMs were developed to avoid these complications and have been proven to be a safer alternative. BSMs, such as hydroxyapatite, β-tricalcium phosphate and calcium sulphate are commonly used synthetic materials in OWHTO [7].

The treatment of osteotomy gaps is still controversial. Han et al. [8] reported no differences when using various types of grafts in the osteotomy gap. Hohmann [9] commented that BSM did not result in higher union rates than the results without graft (WG). Additionally, a systematic review by Lash et al. [10] indicated that BSM had a delayed union rate of 4.5%, higher than the 2.6% for AU. Commercial BSM differs significantly in calcium concentration, particle size and crystallinity, affecting their performance *in vivo* [11,12].

Previous meta-analysis studies contained limited subgroup analysis of BSM and a subgroup analysis of long locking plate (LLP) and short locking plate (SLP) [8,10,13]. Furthermore, previous meta-analysis studies did not include well-designed studies on BSM [10,13].

Hence, we conducted a systematic review and quantitative evaluation of the effectiveness of BSM and BG and WG in OWHTO. Our study assessed the occurrence rate of non-union and complications to provide orthopaedic surgeons with up-to-date information in this area. We hypothesized that BSM combined with a locking plate could better achieve a larger osteotomy gap size effectively. We present the following article in accordance with the PRISMA reporting checklist.

## Methods

This meta-analysis was performed according to the guidelines of the PRISMA statement and the recommendation of the Cochrane Collaboration Group [14].

### Inclusion criteria

The inclusion criteria for the studies were (1) study design: randomized controlled trials (RCTs) and non-randomized studies (NRSs); (2) patients with osteoarthritis or varus knee who underwent HTO; (3) interventions and comparisons: studies compared BSM with AU, AL and WG; and (4) outcomes: primary outcomes including opening gap size, the occurrence rate of non-union. Secondary outcomes including LHFs, knee functional score, infection and Visual Analogue Scale (VAS). Bone union was classified according to the grading systems described by Brosset et al. [15] and Jung et al. [16].

### Exclusion criteria

Unrelated topics, reviews, editorials, letters to the editor, case reports, animal experiments, *in vitro* studies, biomechanics studies and xenograft studies were excluded.

### Search strategy and study selection

According to the guidelines of Preferred Reporting Items for Systematic Reviews and Meta-Analysis (PRISMA) statement, many comprehensive literature databases, including PubMed, Embase, Web of Science, Google Scholar and the Cochrane Library, were searched for studies evaluating BSM filling of the OWHTO osteotomy gap, with a restriction to English articles and no limits on the region and publication type. For our search strategy, the following keywords were used in all fields: “graft” and “high tibial osteotomy” and “knee osteoarthritis”. We utilized the “related articles” function to broaden the search results, and the computer search was supplemented with manual searches of the reference lists for all retrieved studies, review articles and conference abstracts. When multiple reports describing the same population were published, the most recent or complete report was used.

### Data extraction

Data from the included studies were extracted and summarized independently by two authors (author 2 and author 3). The extracted data included the study design, level of evidence, number of participants, mean age, type of BSM, type of BG or WG, functional score, fixation type and mean follow up. Any disagreement was resolved by the adjudicating senior author (corresponding author).

### Methodological and outcomes quality assessment

The Cochrane risk of bias tool (version 1.0) was used to assess the methodological quality of RCTs, while the Risk of Bias in Non-randomized Studies of Interventions (ROBINS-I) tool [17] was used to evaluate risk of bias for NRS. Summary of bias was created by robvis packages of RStudio software (Version 1.4.1106, Camp Pontanezen, Hoboken, NJ). The quality of each outcome was assessed according to the Grades of Recommendation, Assessment, Development and Evaluation (GRADE) Working Group system [18,19].

### Statistical analysis

All the meta-analyses were performed using meta packages of RStudio software (Version 1.4.1106, Camp Pontanezen, Hoboken, NJ). The mean difference (MD) and odds ratio (OR) were used to compare continuous and dichotomous variables, respectively. The inverse-variance method was used for data synthesis. All results were reported with 95% confidence intervals (CIs). Statistical heterogeneity between studies was assessed using the chi-square test with significance set at *p*<.10, and heterogeneity was quantified using the *I*^2^ statistic. If *I*^2^ was <50%, the fixed-effects model was used to pool the effect size; if *I*^2^ was >50%, the random-effects model was used to pool the effect size. Subgroup analyses were conducted according to type of study (RCTs or NRS) and plate (LLP or SLP). Sensitivity analysis was performed by omitting one study at a time to determine the robustness of the pooled results. A funnel plot was generated to assess the publication bias.

## Results

### Literature search results

**Figure 1.**
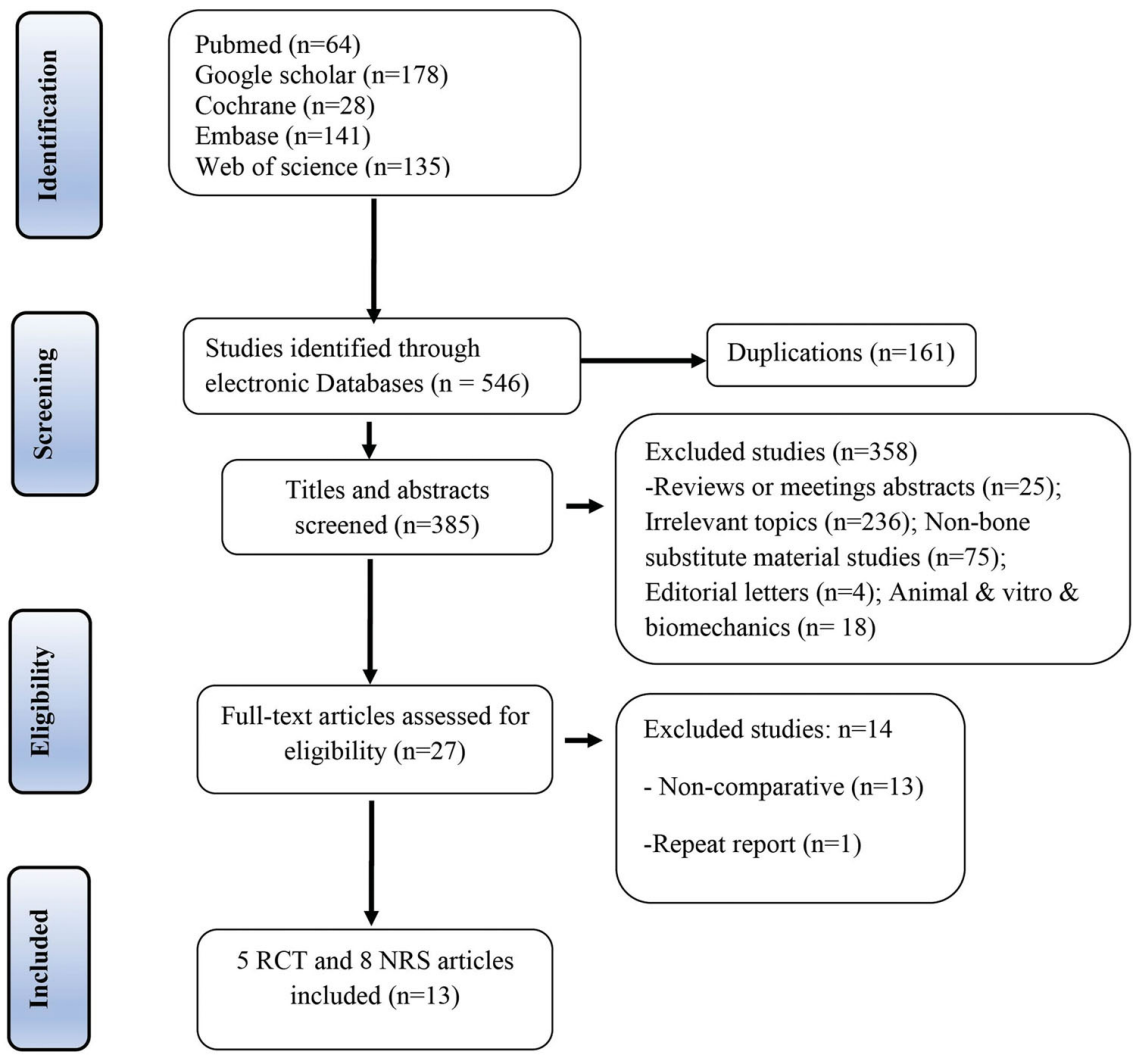
PRISMA flow diagram of studies identified, included and excluded.

During the initial electronic search, approximately 546 studies on OWHTO were identified. After removing duplicates, 385 studies remained. After screening abstract and titles, 27 studies remained. After reading full-text articles, a total of 13 studies were included in this meta-analysis according to the inclusion and exclusion criteria. The flow diagram for the study selection procedure is shown in Figure 1.

### Characters of included studies

Among the included studies, there were five RCTs [20–24] and eight NRS including one prospective study [25], one non-inferiority study [26] and six retrospective studies [16,27–31]. The included studies were conducted between 2010 and 2021 and involved 373 patients treated using BSM, 77 patients treated with AU, 164 patients treated with AL and 155 patients treated with no graft. The average age of the patients ranged from 44 to 61 years. The fixing plates utilized were LLP, SLP, short spacer plate (SSP) and short spacer locked plate (SSLP). The average follow-up period ranged from 6 to 24 months. In a retrospective study by Kim et al. [31], 97 knees were divided into three groups, which were assigned to treat with HA chip, allogenic chip bone (AL) or WG. We separated the three groups into two comparative groups: HA vs. AL and HA vs. WG. The characteristics of the included studies are summarized in Table 1.

**Table 1.** Characteristics of included studies.

Study	Level of evidence	Design	Patients, no.		Mean age (years)		Filler type		Functional score	Fixation type	Mean follow up
			BSM	BG and WG	BSM	BG and WG	BSM	BG and WG			
Dallari et al. [21]	2b	RCT	22	9	46	49	BNMH or BNMH + HDBM	AL	KSS	SSP	12
Lee et al. [23]	2b	RCT	27	27	48.7	52	β-TCP granule	AL	WOMAC/VAS	LLP	12
Jung et al. [16]	3b	NRS	46	48	59.6	61.5	β-TCP	WG	IKDC/Lysholm	LLP	22
Ferner et al. [25]	3b	NRS	19	30	44	50	β-TCP	WG	NA	LLP	24
Gouin et al. [20]	2b	RCT	22	18	51	51	CP wedges	AU	KSS	SLP	45
Hernigou et al. [26]	2b	Non-inferiority clinical trial	17	17	57	51	β-TCP	AU	SF-12/VAS	SLP	6
Lind-Hansen et al. [22]	2b	NRS	15	15	45.6	47.8	Injectable CP	AU	KOOS	SSP	24
Nha et al. [29]	3b	NRS	33	38	55.9	58.3	HA or β-TCP	WG	NA	LLP	24
Lee et al. [27]	3b	NRS	41	53	56.2	56.7	HA chip	AL	KSS, WOMAC	SSLP	12
Drogo et al. [24]	2b	RCT	13	13	56.8	58.1	NHA + AL	AL	WOMAC, KOOS	SLP	60
Jeon et al. [30]	3b	NRS	27	27	54.4	54.6	β-TCP	AU	Lysholm score	LLP	12
Kim et al. [31]	3b	NRS	29	29	53	57.8	HA chip	AL	KSS, WOMAC	LLP	34
Kim et al. [31]	3b	NRS	29	39	53	57.2	HA chip	WG	KSS, WOMAC	LLP	34
Lee et al. [28]	3b	NRS	33	33	50.2	50.3	HA chips	AL	IKDC, KOOS	LLP	24

RCT: randomized controlled trial; NRS: non-randomized study; NA: data not available; KSS: Knee Society Score; IKDC: International Knee Documentation Committee score; VAS: Visual Analogue Scale score; WOMAC: Western Ontario and McMaster Universities Osteoarthritis Index score; KOOS: Knee Injury & Osteoarthritis Outcome; HDBM: human demineralized bone matrix; BNMH: biomimetic nano-structured Mg-hydroxyapatite; β-TCP: beta-tricalcium phosphate; HA: hydroxyapatite; AU: autogenous iliac crest; AL: lyophilized bone chips; CP: calcium phosphate; injectable calcium phosphate cement (Calcibon); SSP: short spacer plate; LLP: long locking plate (TomoFix or OhtoFix); SLP: short locking plate; SSLP: short space locking plate.

**Figure 2.**
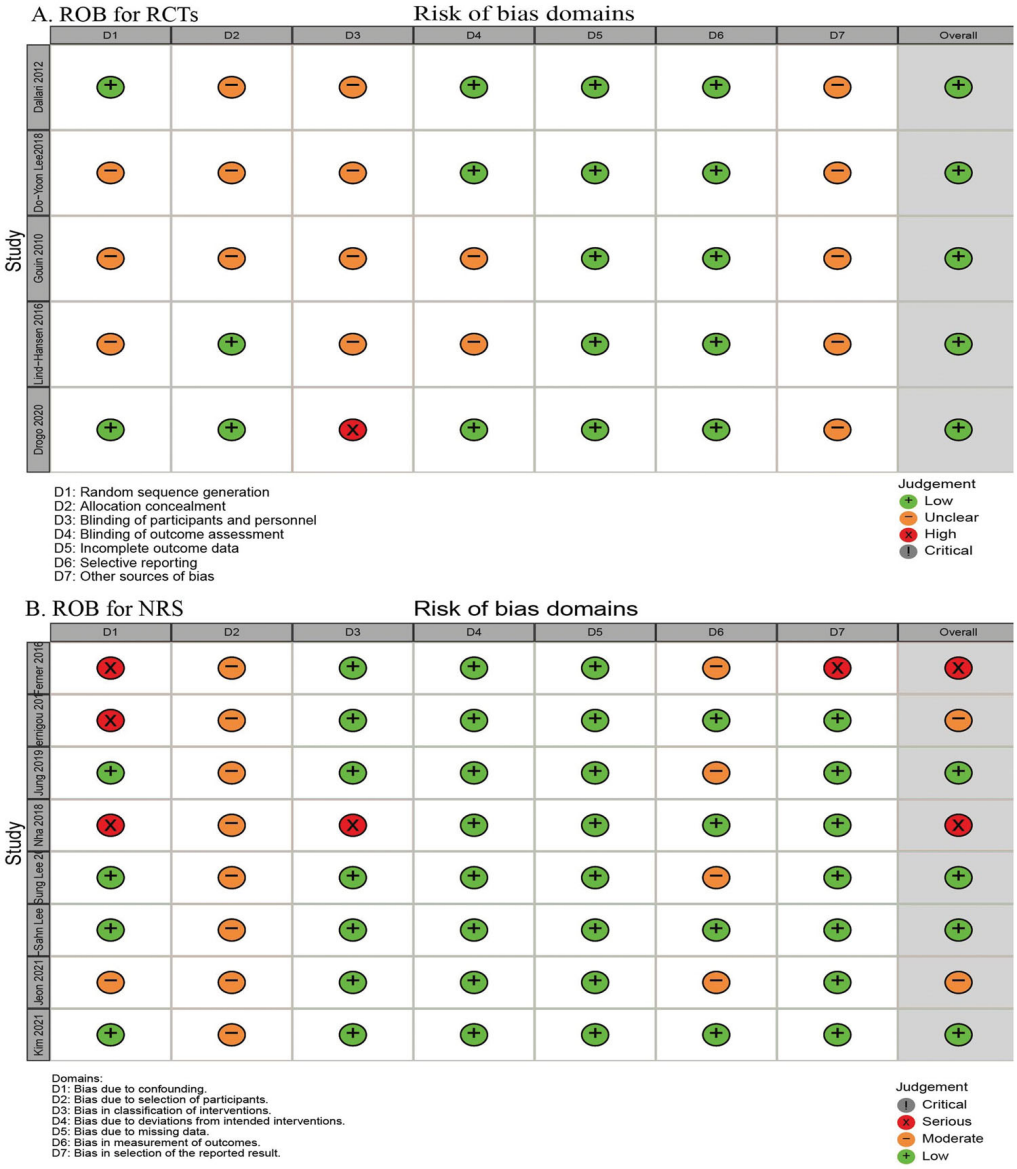
Summary of risk of bias. (A) Risk of bias for RCTs based on the Cochrane Collaboration tool (version 1.0). (B) Risk of bias for NRS based on ROBINS-I tool.

The five RCTs [20–24] reported all experiment results. The studies reported no blinding of participants because of the precise treatment method used for the osteotomy gap in OWTHO. Dallari et al. [21] described random grouping by random number generation. Lin-Hansen et al. [22] reported allocation concealment. Dallari et al. [21], Drogo et al. [24] and Lee et al. [23] reported blinding of the outcomes. We interpreted studies with other biases as low-quality studies. Summary of risk of bias for RCTs is shown in Figure 2(A). For the eight non-RCT studies, the ROBINS-I tool was used to assess the risk of bias, which was evaluated based on bias due to confounding, bias in selection of participants into the study, bias in classification of interventions, deviations from intended interventions, missing data, measurement of outcomes and selection of the reported result. The assessment for each study is shown in the Supplementary material (supplementary table). Summary of risk of bias for the eight NRS is shown in Figure 2(B).

### Clinical outcomes

Due to high heterogeneity among the included studies, random effects model was used to synthesize all outcomes.

### Primary outcomes

#### Osteotomy gap size

**Figure 3.**
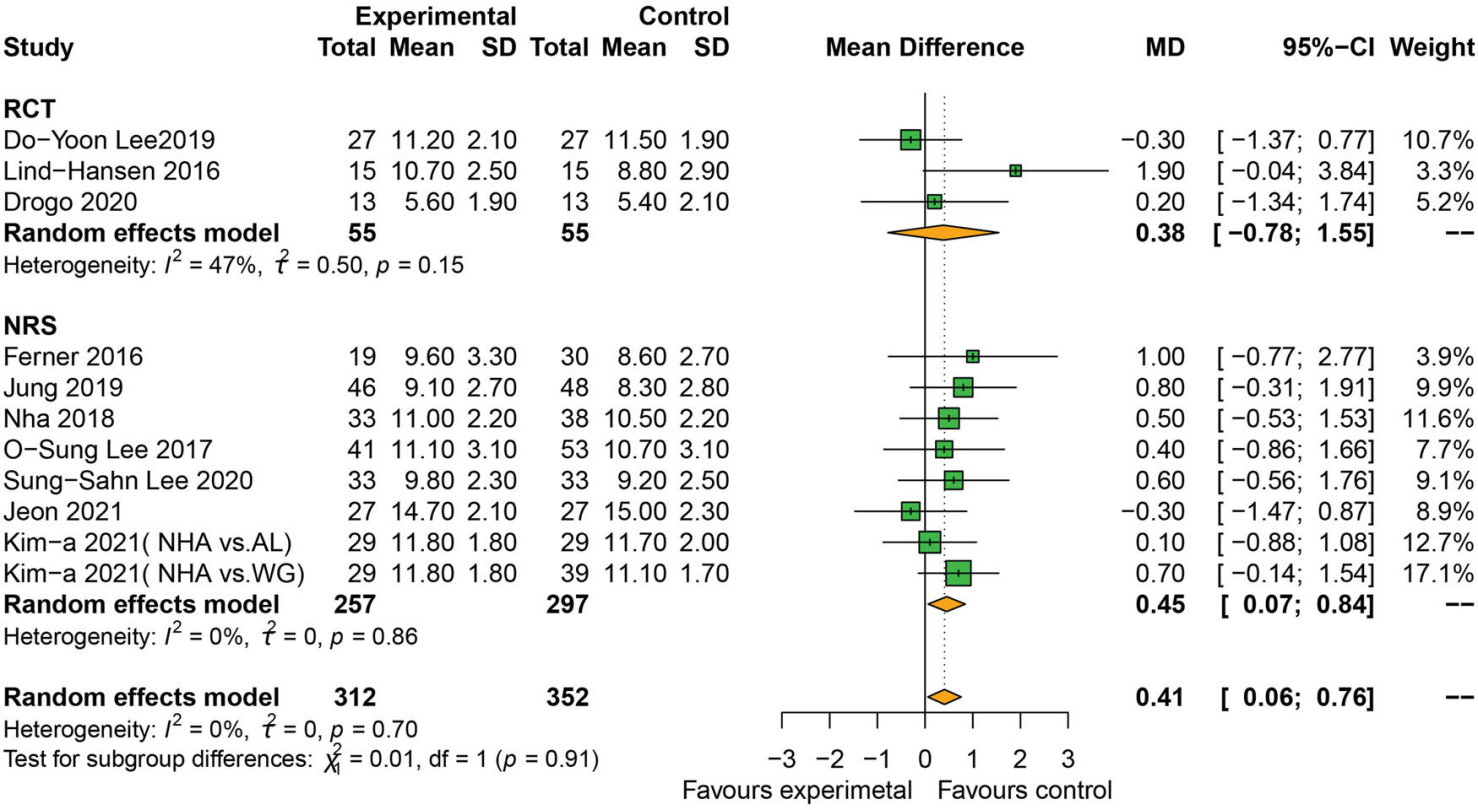
Forest plot of comparison: osteotomy gap size between BSM and BG and WG groups after OWHTO. OWHTO: opening wedge high tibial osteotomy; KOA: knee osteoarthritis; CI: confidence interval; SD: standard deviation; BSM: bone substitute material; BG: bone graft; WG: without graft.

Osteotomy gap size was reported in 10 studies including 11 comparative groups [16,22–25,27–31] (312 patients treated with BSM and 352 treated with BG and WG). The overall pooled effect suggested there was a slightly larger osteotomy gap size in the BSM group when compared to the BG and WG group (MD: 0.41 mm, 95%CI: [0.06, 0.76], *p*=.02, *I*^2^=0%), as revealed by the random effects model (Figure 3). The pooled effect of RCTs [22–24] showed there was no significant difference between the two groups as revealed by random effects model (MD: 0.38 mm, 95%CI: [–0.78, 1.55], *p*=.52, *I*^2^=47%). The pooled effect from NRS [16,25,27–31] showed the osteotomy gap size was 0.45 mm larger in BSM group than BG and WG group, with significant difference as revealed by random effects model (95%CI: [0.07, 0.84], *p*=.02, *I*^2^=0%) (Figure 3).

#### Bone non-union

**Figure 4.**
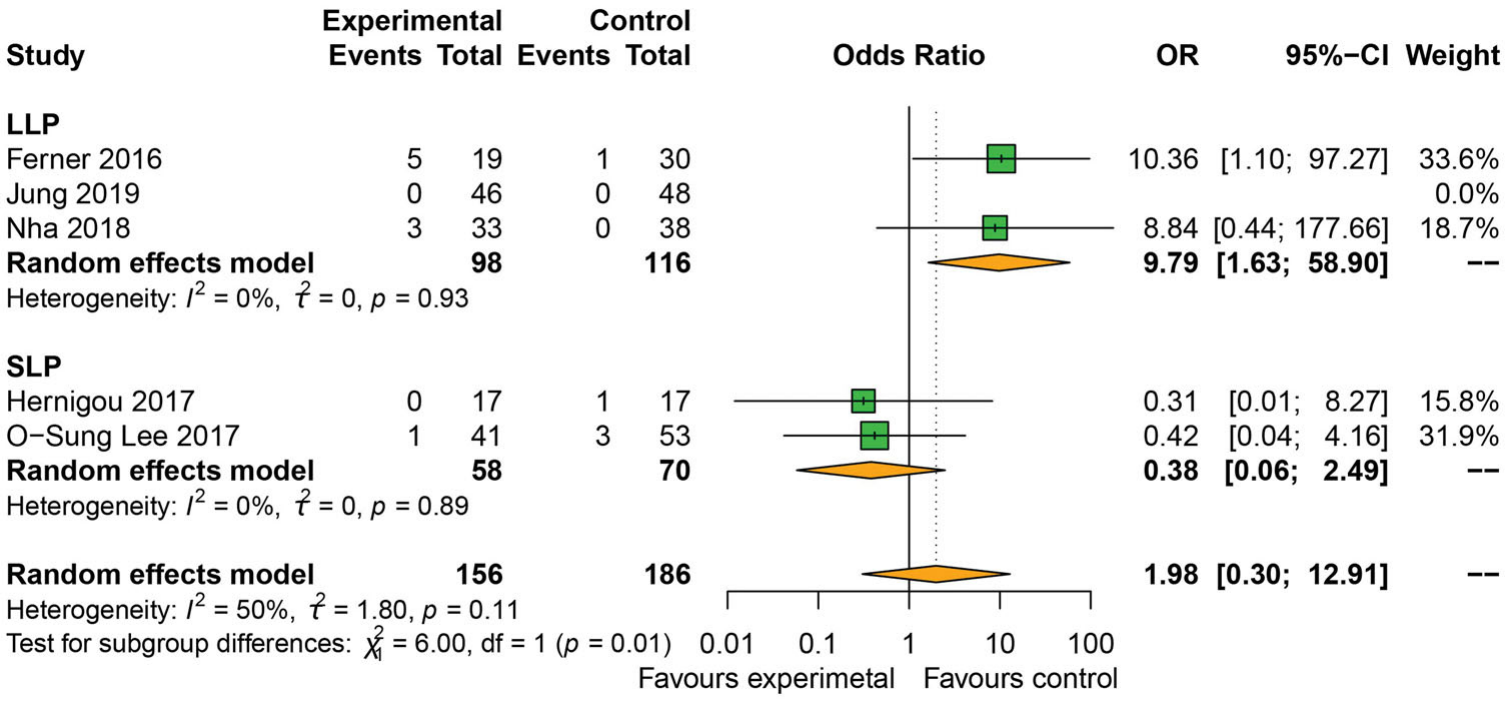
Forest plot of the incidence of non-union in osteotomy gap filled with BSM and BG or WG. Subgroup analysis conducted by plate types (LLP and SLP). OWHTO: opening wedge high tibial osteotomy; KOA: knee osteoarthritis; CI: confidence interval; SD: standard deviation; BSM: bone substitute material; BG: bone graft; WG: without graft.

Five NRS studies reported bone non-union [16,25–27,29], and subgroup analysis was conducted by graft type. There was no significant difference between the BSM group and the BG and WG group in OWHTO in the main analysis (OR: 1.98, 95%CI: [0.30, 12.91], *p*=.48). In the LLP subgroup, pooled data from Ferner et al. [25], Jung et al. [16] and Nha et al. [29], who assessed non-union between the BSM and control groups (98 patients treated with BSM and 116 treated with BG and WG), showed an OR of 9.79, with significant difference (95%CI: [1.63, 58.90], *p*=.01). However, in the SLP subgroup, pooled data from Hernigou et al. [26] and Lee et al. [27], who assessed non-union in BSM and WG groups (58 patients treated with BSM and 70 treated with BG and WG), revealed no significant difference between the BSM and the BG and WG groups (OR: 0.38, 95%CI: [0.06, 2.49], *p*=.31) (Figure 4(B)).

### Secondary outcomes

#### LHFs

**Figure 5.**
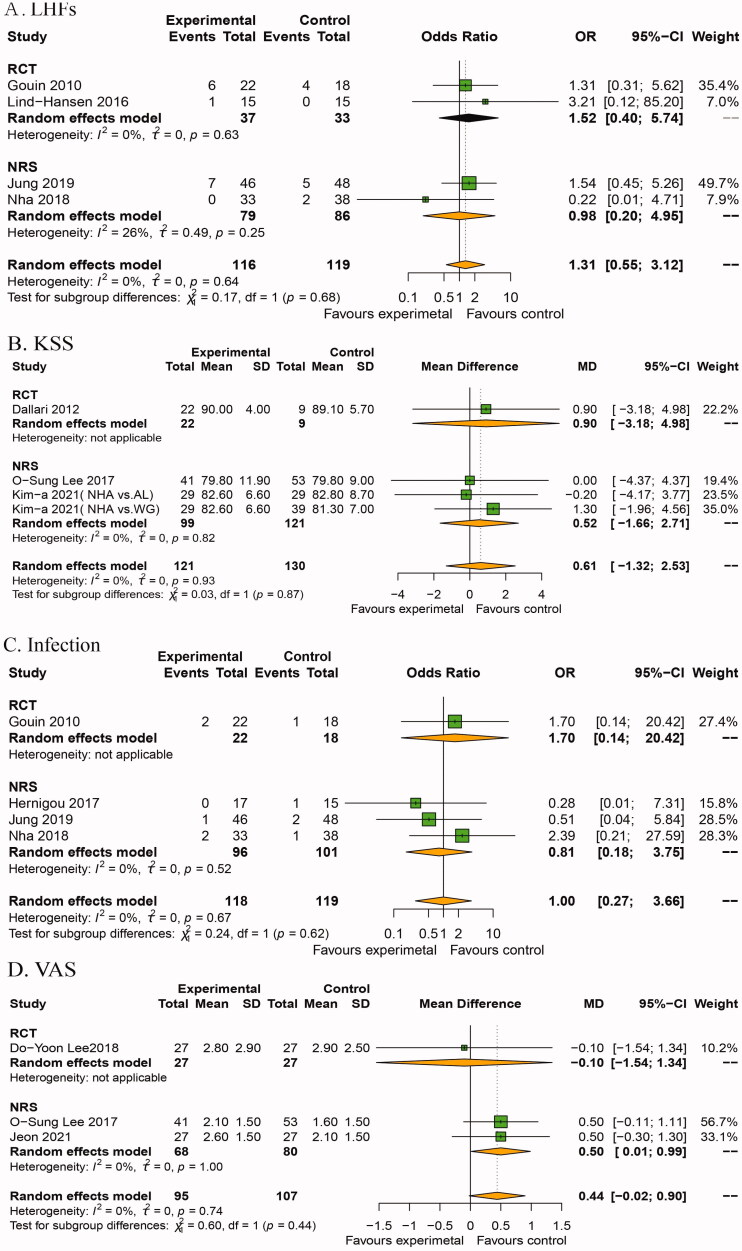
Forest plot of secondary outcomes between BSM and BG and WG groups after OWHTO. (A) LHFs, (B) KSS, (C) infection and (D) VAS. OWHTO: opening wedge high tibial osteotomy; KOA: knee osteoarthritis; CI: confidence interval; SD: standard deviation; BSM: bone substitute material; BG: bone graft; WG: without graft; LHFs: lateral hinge fracture.

Two RCTs from Gouin et al. [20] and Lind-Hansen et al. [22] and two NRS from Jung et al. [16] and Nha et al. [29] reported the incidence of LHFs. The overall pooled effect showed there was no significant difference between the two groups in the occurrence of LHFs (OR: 1.31, 95%CI: [0.55, 3.12], *p*=.54). In subgroup analysis by type of study, the pooled effect of RCT and NRS was 1.52 (95%CI: [0.40, 5.74], *p*=.54) and 0.98 (95%CI: [0.20, 4.95], *p*=.98), respectively, without significant difference (Figure 5(A)).

#### KSS

One RCT from Dallari et al. [21] and two NRS including three comparative groups from Lee et al. [27] and Kim et al. [31] reported KSS. The overall pooled effect suggested no significant differences existing between BSM and WG groups in OWHTO postoperatively (MD: 0.61, 95%CI: [–1.32, 2.53], *p*= .54). In subgroup analysis, the MD reported by RCT and NRS was 0.90 (95%CI: [–3.18, 4.98], *p*=.67) and 0.52 (95%CI: [–1.66, 2.71], *p* = 1.00), respectively, no significant difference existing between the two groups (Figure 5(B)).

### Infection

One RCT form Gouin et al. [20] and three NRS from Hernigou et al. [26], Jung et al. [16] and Nha et al. [29] reported the incidence of infection (118 patients treated with BSM and 119 treated with BG and without a graft). The overall pooled effect showed there was no significant difference between the BSM and the BG and WG groups in terms of infection (OR: 1.00, 95%CI: [0.27, 3.66], *p*= 1.00) (Figure 5(C)). In subgroup analysis by type of plate, the pooled effect of RCT and NRS showed an OR of 1.70 and 0.81, respectively, without significant difference (Figure 5(C)).

#### VAS

One RCT from Lee et al. [23] and two NRS from Lee et al. [27] and Jeon et al. [30] reported VAS score (95 patients treated with BSM and 107 treated with BG or WG). Overall pooled effect revealed no significant difference between BSM and control groups (MD: 0.44, 95%CI: [–0.02, 0.90], *p*= .06) in terms of VAS. Pooled effect from RCT subgroup showed an MD of −0.10, without significant difference (95%CI: [–1.54, 1.34], *p*=.89); however, the pooled effect from NRS subgroup showed an MD of 0.50, with a significant difference (95%CI: [0.01, 0.99], *p*=.04) (Figure 5(D)).

#### Evidence of GRADE quality

The GRADE quality of evidence assessments is summarized in Table 2. Overall, the quality of evidence for outcomes was low.

**Table 2.** Summary of findings.

Quality assessment						Summary of Findings			
Outcomes	Risk of bias	Inconsistency	Indirectness	Imprecision	Publication bias	No. of patients		Mean difference or relative effect (95%CI)	Overall quality of evidence
						BSM	Control		
Opening gap size RCT (critical outcome)									
110 (3 studies)	No serious	Serious^c^	No serious indirectness	Very serious	Reporting bias strongly suspected^f^	55	55	0.38 (–0.78 to 1.55)	⊕⊕ Low
Opening gap size NRS (critical outcome)									
554 (7 studies)	Serious^a^	No serious inconsistency	No serious indirectness	No serious imprecision	Undetected	257	297	0.45 (0.07–0.84)	⊕⊕ Low
Bone non-union LLP (NRS, critical outcome)									
214 (3 studies)	Serious^b^	No serious inconsistency	No serious indirectness	Very serious^d^	Reporting bias strongly suspected^f^	98	116	9.79 (1.63–58.90)	⊕⊕ Low
Bone non-union SLP (NRS, critical outcome)									
128 (2 studies)	Very serious^b^	No serious inconsistency	No serious indirectness	Very serious^d^	Reporting bias strongly suspected^f^	58	70	0.38 (0.06–2.49)	⊕⊕ Low
KSS – RCT (important outcome)									
31 (1 study)	No serious risk of bias	Not applicable^e^	Not applicable^e^	Not applicable^e^	Reporting bias strongly suspected^f^	22	9	0.9 (–3.18 to 4.90)	Not applicable
KSS – NRS (important outcome)									
220 (2 studies)	Serious^a^	No serious inconsistency	No serious indirectness	Serious^d^	Reporting bias strongly suspected^f^	99	121	0.52 (–1.66 to 2.71)	⊕⊕ Low
LHFs – RCT (important outcome)									
70 (2 studies)	No serious risk of bias	No serious inconsistency	No serious indirectness	Very serious^d^	Reporting bias strongly suspected^f^	37	33	1.52 (0.40–5.74)	⊕⊕ Low
LHFs – NRS (important outcome)									
165 (2 studies)	Serious^a^	No serious inconsistency	No serious indirectness	Very serious^d^	Reporting bias strongly suspected^f^	79	86	0.98 (0.20–4.95)	⊕⊕ Low
Infection – RCT (important outcome)									
40 (1 study)	No serious risk of bias	Not applicable^e^	Not applicable^e^	Not applicable^e^	Reporting bias strongly suspected^f^	22	18	1.7 (0.14–20.42)	Not applicable
Infection – NRS (important outcome)									
197 (3 studies)	Serious^a^	No serious inconsistency	No serious indirectness	Serious^d^	Reporting bias strongly suspected^f^	96	101	0.81 (0.18–3.75)	⊕⊕ Low
VAS – RCT (important outcome)									
54 (1 study)	No serious	Not applicable^e^	Not applicable^e^	Not applicable^e^	Reporting bias strongly suspected^f^	27	27	–0.10 (–1.54 to 1.34)	Not applicable
VAS – NRS (important outcome)									
148 (2 studies)	Serious^a^	No serious inconsistency	No serious indirectness	Very serious^d^	Reporting bias strongly suspected^f^	68	80	0.50 (0.01–0.99)	⊕⊕ Low

^a^ The quality of studies limited to confounding, selection of participants and measurement of outcomes.

^b^ The quality of studies limited to confounding, classification of intervention, selection of participants and measurement of outcomes.

^^c^^Significant heterogeneity among included studies (*I*^2^=74%).

^d^ Wide CIs around estimated effect, total sample size < 400.

^e^ Only one study included.

## Discussion

OWHTO improved the postoperative function score significantly and relieved pain in KOA patients by shifting the mechanical axis from the medial compartment to the healthy lateral compartment to decrease the load and progression of osteoarthritis in the lower extremity. This study aimed to evaluate the efficacy and safety of BSM in OWHTO. An osteotomy gap with a larger size may achieve bone union for a longer period, possibly associated with higher rates of complications such as LHFs and loss of correction. Delayed union and non-union of the osteotomy gap are common complications in OWHTO. Our meta-analysis was performed with the radiological and clinical outcomes of BSM, especially the occurrence rate of non-union, compared with BG and WG.

This study demonstrated the osteotomy gap size in the BSM group (>10 mm) was 0.52 mm larger than that in the BG and WG group (mean 10 mm). BSM application may become an alternative option for the larger osteotomy gaps in OWHTO (Figure 3). According to previously published literature, the osteotomy gap size was 10.3 mm with the synthetic materials, 9.4 mm with AL, 9.8 mm with AU and 10.2 mm with no filler. Additionally, a bone filler is recommended for osteotomy gap sizes > 10mm [13,30,32]. However, the cut-off value for each type of bone filler is still unclear. In the present study, the 95%CI for the effect was very close to the zero effect line and would change if sensitivity analysis was conducted by withdrawing one included study. Hence, surgeons should be cautious when using BSM in osteotomy gap > 10 mm.

The appropriate bone treatments for filling the osteotomy gap are still controversial [8,10,33]. Zorzi et al. [34] indicated no significant bone union in the AU group (12.4 weeks) and the WG group (13.7 weeks) with a non-locking Puddu plate. Moreover, Fucentese et al. [35] demonstrated no functional advantage between AU (*n*= 15) and WG (*n*= 25) groups at 3 or 12 months postoperatively, and Brosset et al. [15] reported that the bone union occurred at 4.5 months on average with locking plate fixation using WG treatment in OWHTO.

Gaasbeek et al. [36] reported that β-tricalcium phosphate used in osteotomy gaps results in an excellent bone union combined with locking plate technology. Jung et al. [16] reported that 17 patients with osteotomy gaps >10 mm treated using β-tricalcium phosphate graft achieved bone union at 8.6 ± 3.6 months compared with 8.8 ± 3.4 months in the WG group (*n*= 13). Jung et al. [16] found that AU graft with β-tricalcium phosphate resulted in the fastest radiological bone union and best clinical scores at six months of follow-up.

Lee et al. [23] reported that the β-tricalcium phosphate granule group achieved bone union in comparison with the AL chip grafts group at 6 and 12 months postoperatively. Hydroxyapatite and β-tricalcium phosphate ceramics are manufactured in various forms, such as granules and porous blocks, which are attractive alternatives for the osteotomy gap [11]. From the included studies, postoperative biopsy indicated that the application of BSM is safe. Dallari et al. [21] reported that nanocomposites (DBSint^®^) were safe and effective as lyophilized bone chips in OWHTO, and no acute or chronic infection was found surrounding BSM grafts.

Some studies indicated that BSM grafts could not be completely resorbed [12]. Lee et al. [28] reported remnant hydroxyapatite and mature bone identified via biopsy of haematoxylin–eosin staining. Ferner et al. [25] also said residual β-tricalcium phosphate in the osteotomy gap. Aryee et al. [7] reported prominent remnants of hydroxyapatite/β-tricalcium phosphate in the osteotomy gap. Putnis et al. [37] reported a wedge of biphasic calcium phosphate (BCP) combined with a locking plate provided good clinical outcomes and remained radiographically visible. These clinical outcomes may provide useful insights for the use of BSM in the osteotomy gap to rival the AU in the future.

This study revealed that the non-union in the osteotomy gap of the BSM group was the same as that observed in the BG group. Hence, the use of BSM in OWHTO is safe and efficient based on the same complications. Furthermore, potential advantages include less blood loss when the osteotomy gap is filled with BSM.

The AU graft can be usually harvested from the iliac crest [7], the ipsilateral medial femoral condyle [16] and two adjacent cut surfaces of local bone [22]. The disadvantages of an iliac crest harvest include the surgical donor site, possible postoperative pain, blood loss, haematoma, infection, fracture, neurovascular injury and longer operative time [11]. An autologous iliac crest graft was recommended for KOA patients with morbidities such as obesity, smoking and an opening angle greater than 10° [7]. Hernigou et al. [26] reported that β-tricalcium phosphate in the osteotomy gap (*n*= 17) resulted in bone union compared with the results in the AU graft group (16 among 17) at 12 weeks.

Our study hypothesized that the application of the locking plate improved the clinical and radiological results. The occurrence rate of non-union of the osteotomy gap in the WG group (<10 mm) with the locking plate was lower than that in the BSM group (Figure 4(A)). The occurrence rate of non-union in the WG group with LLP was lower than that in the BSM group (Figure 4(B)). An RCT study by Nha et al. [29] reported 93.9% of BSM consisting of hydroxyapatite and β-tricalcium phosphate along with LLP showed bone union over zone 3 at two years. The control group (WG) showed good clinical and radiological results without correction loss at two years and a more significant incorporation than that of the BSM group during follow-up [13]. Interestingly, the WG subgroup included the same studies as the LLP subgroup. The possible explanation was that the LLP has angular stability, maintains the stability of the osteotomy gap, promotes bone union and avoids loss of correction angle for OWHTO without void filling [38–41].

The stability of a locking plate is better than that of a non-locking plate. Kuremsky et al. [42] reported that freeze-dried cortical, cancellous structural grafts (AL, *n*= 51) with a short non-locking plate (Arthrex, Munich, Germany) are associated with sixfold higher failure rates compared with the use of iliac crest BG (*n*= 19). It should be noted that structural grafts are considered unnecessary with the development of modern locking plates [8,43]. Therefore, a fragmented cancellous or wedge-shaped cancellous bone, combined with locking plate technology, can achieve better bone union than that of structural AL grafts.

In a biomechanics study, Takeuchi et al. [44] reported that the use of β-tricalcium phosphate wedges with the TomoFix plate improved the initial axial and possibly rotational stability compared with no graft filling. Belsey et al. [45] reported that the use of graft materials in OWHTO resulted in superior material properties compared with the use of no graft with LLP (Activmotion 2). In contrast, Floerkemeier et al. [4] demonstrated that BSM with LLP (*n* = 533) caused a fourfold higher risk of complications in OWHTO patients than in WG patients.

The limitations of LLP include some abnormal subcutaneous sensations and the high cost. Thus, SLP is a novel design with increased stability, lower price and more comfort to patients. This study indicated no significant differences between BSM and BG and WG groups with SLP in the occurrence rate of non-union. Turkmen et al. [46] reported WG with an average size of 11.07 mm achieved successful bone union at 12.8 weeks in 41 knees with SLP. Hernigou et al. [47] demonstrated BSM could achieve osseointegration combined with SSP (Limmed^®^), with immediate full weight-bearing compared with non-locking plates without full weight-bearing. Furthermore, Dallari et al. [21] reported no significant difference in the loss of correction between BSM and AL with SLP.

There is another controversy in clinical practice. β-Tricalcium phosphate manufactured by different companies worldwide has different characteristics. Ferner et al. [25] reported that β-tricalcium phosphate graft (Actifuse Granules^®^) had 26% non-union (five out of 19 cases) compared with 3.3% non-union in the WG graft group (one case out of 30). Further, Gouin et al. [20] reported that six out of 22 patients lost the correction in the BMCaPh calcium phosphate wedges group, resulting in three early surgical revisions compared to only one among the 18 patients in the AU group with SLP.

There were no significant differences between the BSM and the BG and WG groups in terms of LHFs, functional score, infection and VAS scores (Figure 5). LHFs are associated with the delayed bone union and loss of correction, especially Takeuchi type II fractures [48]. The loss of correction was defined as over 4° by comparing the immediate postoperative image with the final follow-up radiographic findings [34]. The incidence of LHFs, determined using computed tomography, was 13.8% higher than the detection rates by plain radiographs (9.2%) [49]. Gouin et al. [20] reported that there were six patients with LHFs in the BMCaPh calcium phosphate wedges group with SLP compared to four patients in the AU group. Pooled data from Nha et al. [29] indicated that two patients with LHFs with LLP in the BSM group showed union in zones 3 and 4 at two years without a loss of correction. Perhaps the stability of LLP will contribute to bone union healing in the BSM group.

We acknowledge there are some limitations in our meta-analysis. First, the type of studies in our final studies was not uniform, which would impact on the reliability of our results due to the methodological heterogeneity. Second, inadequate random sequence generation and blinding tend to increase the risk of detection bias. Third, different manufacturers produce different types of BSM with unusual bone healing abilities. This was another source of heterogeneity and affected the reliability of the evaluation of bone union. Four, most studies were conducted in a single centre and had a small sample size. Fifth, due to the small number of RCT articles included, there was no significance in the sensitivity analysis and funnel plot to assess publication bias. Finally, the included studies were carried out using fixation plates with different fixation properties, different BSM and surgical expertise. The experience of the surgeon could influence the outcomes. We applied multiple strategies to identify studies and strict criteria to include and evaluate the methodological quality of the reviews, and subgroup and sensitivity analysis to minimize the heterogeneity.

Our systematic review showed that BSM combined with locking plate techniques offers a safe and efficient alternative option in OWHTO for osteotomy gaps slightly larger than 10 mm. However, BSM grafts as they are not entirely resorbed through biopsy histology. The osteotomy gaps with an average gap size of 10 mm achieve bone union without a graft using an LLP. Given the inherent limitations of the included studies, future well-designed RCTs are required to verify the findings of this meta-analysis.

## Supplementary Material

Supplemental Material

## Data Availability

The data synthesized and presented in the results section have been well referenced as an update systematic review article. However, raw data (in excel sheet) used in the statistical analysis will be made available on request through the corresponding author (Junting Liu).

## Abbreviations

OWHTO: opening wedge high tibial osteotomy
KOA: knee osteoarthritis
CI: confidence interval
SD: standard deviation
BSM: bone substitute material
BG: bone graft
WG: without graft
AU: autograft
AL: allograft
LLP: long locking plate
SLP: short locking plate
LHF: lateral hinge fracture
KSS: Knee Society Score
VAS: Visual Analogue Scale
