# Supplementary material for: Effectiveness of bone substitute materials in opening wedge high tibial osteotomy: a systematic review and meta-analysis
Source: Ann Med. 2022 Feb 15;54(1):565–77. doi: 10.1080/07853890.2022.2036805 (PMC8856078; doi:10.1080/07853890.2022.2036805)
Supplement: Supplemental Material [file IANN_A_2036805_SM2991.docx]

**Supplementary Table. Risk of bias summary for non-RCTs Non-randomized Study of Intervention (NRS): review authors’ judgements about each risk of bias item for each included study.**

| **STUDY TYPE: NRS** | | | | | | | |
| --- | --- | --- | --- | --- | --- | --- | --- |
| **AUTHOR (YEAR)** | **BIAS DUE TO CONFOUNDING** | **BIAS IN SELECTION OF PARTICIPANTS INTO STUDY** | **BIAS IN CLASSIFICATION OF INTERVENTIONS** | **BIAS DUE TO DEVIATIONS FROM INTENDED INTERVENTION** | **BIAS DUE TO MISSING DATA** | **BIAS IN MEASUREMENT OF OUTCOMES** | **BIAS IN SELECTION OF THE REPORTED RESULT** |
| Ferner 2016 | Serious  The baseline between the two groups was similar. However, patients enrolled in the no augmentation group were prior to synthetic augmentation group. | Moderate  Patients were assigned by date of operation. | LOW  With or without augmentation in OWHTO was well defined interventions. | LOW  The co-intervention was same among the two groups. | LOW  No losses to follow up. | MODERATE  No masking strategy specified | SERIOUS  Postoperative correction angle was only performed in patients with suspicious loss of correction clinically or on plain radiographs of the knee. |
| Hernigou 2017 | Serious  Four patients had bilateral interventions, and the rest had unilateral interventions. Not enough information for baseline similarity judgement. | Moderate  Subject were retrospectively reviewed. | LOW  No filler or filled with β-TCP are well-defined interventions. | LOW  Co-interventions were balanced across intervention groups. | LOW  No losses to follow up. | LOW  The radiographs images were evaluated separately by two investigators and re-evaluated 2 months later. | LOW  All of the indicators mentioned in the methodology are reported |
| Jung 2019 | LOW  The baseline between the two groups was similar. | Moderate  Subject were retrospectively reviewed. | LOW  Autologous Graft and β-TCP are well-defined interventions. | LOW  Co-interventions were balanced across intervention groups. | LOW  No losses to follow up. | MODERATE  No masking strategy specified | LOW  All of the indicators mentioned in the methodology were reported |
| Nha 2018 | Serious  Eight patients had bilateral interventions, and the rest had unilateral interventions. | Moderate  Subject were retrospectively reviewed. | Serious  Although bone void filler was well-defined, the material was not the same, 23 knees with HA and 10 knees withβ-TCP. | LOW  Co-interventions were balanced across intervention groups. | LOW  No losses to follow up. | LOW  The measurement of bone union area was performed by two independent observers who were blinded to other reviewers and their own prior measurements. | LOW  All of the indicators mentioned in the methodology were reported. |
| Lee 2017 | LOW  The baseline between the two groups was similar. | MODERATE  Subject were retrospectively reviewed. | LOW  Allogenous bone chips or HA chips were well defined interventions. | LOW  Co-interventions were balanced across intervention groups | LOW  No losses to follow up. | MODERATE  No masking strategy specified | LOW  All of the indicators mentioned in the methodology were reported. |
| Lee 2020 | LOW  The baseline between the two groups was adjusted by propensity score matching. | MODERATE  Subject were retrospectively reviewed. | LOW  Allogenous bone chips or HA chips were well defined interventions. | LOW  Co-interventions were balanced across intervention groups. | LOW  No losses to follow up. | LOW  All radiographic outcomes were independently determined by two orthopedic surgeons. | LOW  All of the indicators mentioned in the methodology were reported. |
| Jeon 2021 | Moderate  The baseline between the two groups was similar. However, the choice of BSM was made according to the surgeon’s preference. | MODERATE  Subject were retrospectively reviewed. | LOW  Allogenous bone chips or β-TCP granules were well defined interventions. | LOW  Co-interventions were balanced across intervention groups. | LOW  No losses to follow up. | MODERATE  No masking strategy specified | LOW  All of the indicators mentioned in the methodology were reported. |
| Kim 2021 | LOW  The baseline between the three groups was similar. | MODERATE  Subject were retrospectively reviewed. | LOW  The intervention was clearly defined among the participants. | LOW  Co-interventions were balanced across intervention groups. | LOW  No losses to follow up. | LOW  All clinical and radiographic outcomes were independently determined by two orthopedic surgeons. | LOW  All of the indicators mentioned in the methodology were reported. |
